# Quality of life, daily functioning, and symptoms in hypothyroid patients on thyroid replacement therapy: A Dutch survey

**DOI:** 10.1016/j.jcte.2024.100330

**Published:** 2024-02-02

**Authors:** Ellen Molewijk, Eric Fliers, Koen Dreijerink, Ad van Dooren, Rob Heerdink

**Affiliations:** a University of Applied Sciences Utrecht, Utrecht, the Netherlands; b Amsterdam University Medical Centers, Location Academic Medical Center Amsterdam, Department of Endocrinology and Metabolism, Amsterdam Gastroenterology, Endocrinology & Metabolism, the Netherlands; c Amsterdam University Medical Centers, Location VU University, Department of Endocrinology and Metabolism, Amsterdam Gastroenterology, Endocrinology & Metabolism, the Netherlands; d Utrecht Institute for Pharmaceutical Sciences, Utrecht, the Netherlands

**Keywords:** Quality of life, Daily functioning, Hypothyroidism-associated symptoms, Hypothyroid patients, Persisting complaints, Thyroid serum parameters

## Abstract

**Objective:**

To explore the nature and extent of possible residual complaints among Dutch hypothyroid patients using thyroid replacement therapy, we initiated a comprehensive study measuring health-related quality of life (QoL), daily functioning, and hypothyroidism-associated symptoms in patients and control persons.

**Methods:**

An online survey measuring thyroid-specific QoL (ThyPRO), daily functioning, and hypothyroidism-associated symptoms (ThySHI) was distributed among treated hypothyroid patients and control individuals. The advertising text was formulated in an open-ended manner. Patients also provided their most recent thyroid blood values and their thyroid medication.

**Results:**

There was a large-sized impairment of QoL (Cohen’s d = 1.04, +93 % ThyPRO score) in hypothyroid patients on thyroid replacement therapy (n = 1195) as compared to controls (n = 236). Daily functioning was significantly reduced i.e., general health (-38 %), problems with vigorous- (+64 %) and moderate activities (+77 %). Almost 80 % of patients reported having complaints despite thyroid medication and in-range thyroid blood values, with 75 % expressing a desire for improved treatment options for hypothyroidism (total n = 1194). Hypothyroid patients experienced 2.8 times more intense hypothyroidism-associated symptoms than controls (n = 865, n = 203 resp). Patients' median reported serum concentrations were: TSH 0.90 mU/L, FT4 17.0 pmol/L, and FT3 2.67 pmol/L, with 52 % having low T3 levels (<3.1 pmol/L). The QoL was not found to be related to age, sex, BMI, menopausal status, stress, serum thyroid parameters, the origin and duration of hypothyroidism, the type of thyroid medication, or the LT4 dose used.

**Conclusions:**

Our study revealed major reductions in quality of life and daily functioning, and nearly three times more intense hypothyroidism-associated symptoms in treated hypothyroid patients as compared to controls, despite treatment and largely in-range serum TSH/FT4 concentrations. The QoL was not associated with serum thyroid parameters. We recommend future research into the origin of persisting complaints and the development of improved treatment modalities for hypothyroidism.

## Highlights

• Hypothyroid patients on thyroid replacement therapy (n = 1000–1200):
• had large-sized impairments of quality of life (QoL) *vs* controls,
• had significantly reduced daily functioning *vs* controls (n = 240),
• had almost three times more hypothyroid-associated symptoms *vs* controls.
• Serum thyroid parameters and subject background variables were not related to the QoL.
• Comorbidities only accounted for 14% of the patient-control QoL difference

## Graphical abstract

**Figure:**
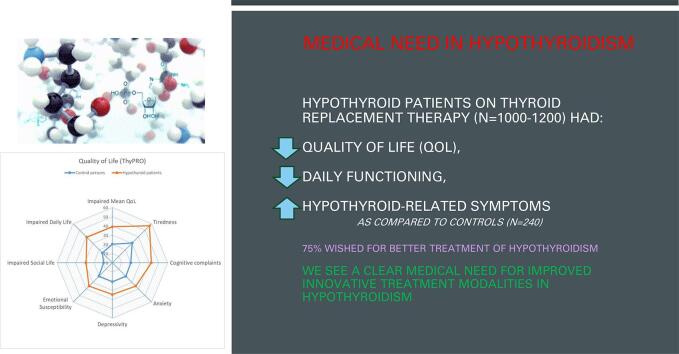

## Introduction

Primary hypothyroidism is a common endocrine disorder with a prevalence of 3.6–5.1 % in adults, occurring 4–7 times more often in females [1]. The condition is mostly due to Hashimoto’s autoimmune disease, is iatrogenic after radioactive iodine treatment for Graves’ disease or after surgery for (non–) malignancy, or is congenital. Current European, American, and British guidelines advise mostly lifelong thyroid hormone replacement therapy with synthetic levothyroxine (LT4) as the standard treatment [2], [3], [4]. The treatment aim is to achieve serum thyroid stimulating hormone (TSH) and free thyroxine (FT4) concentrations within predefined reference ranges [2], [3]. The rationale for using LT4 monotherapy assumes that local conversion into liothyronine (LT3), the active thyroid hormone, would restore euthyroidism in organs and tissues. The current definition of euthyroidism, however, is based on serum TSH/FT4 values only.

A substantial proportion of hypothyroid patients (5 %-15 %) suffers from persisting symptoms despite LT4 treatment and serum TSH/FT4 levels within the reference range [1], [5], [6] Complaints of patients include fatigue, tiredness, reduced vitality, hypothyroidism-associated symptoms, problems with concentration and cognition, and reduced well-being, mood, and reduced health-related quality of life (QoL) [7], [8], [9], [10], [11]. Hence, hypothyroid patients have expressed dissatisfaction with LT4 treatment and felt their complaints were not taken seriously by caregivers [5], [7], [10].

Health risks that have been reported to be associated with suboptimal treatment for hypothyroidism may include cardiovascular disease, obesity, depression, pulmonary consequences, (female) fertility problems, and possibly dementia [12], [13], [14], [15], [16]. Since hypothyroid patients on LT4 treatment may have persisting complaints, use more beta-blockers, statins and antidepressants, have reduced resting energy expenditure (REE), and have reduced exercise tolerance and physical performance capacity, one could question the adequacy of LT4 replacement treatment to restore euthyroidism across the entire body [17], [18], [19]. Despite these findings, LT4 remains the mainstay in the treatment guidelines for hypothyroidism.

To explore the nature and extent of possible residual complaints among Dutch hypothyroid patients using thyroid replacement therapy, we initiated a comprehensive study measuring health-related QoL, daily functioning, and hypothyroidism-associated symptoms in patients and control persons. Patients were additionally asked to respond to daily life statements and report their most recent thyroid blood values, thyroid medication, and past symptoms. The relation between the QoL *vs* background variables (age, sex, menopausal state, body weight, and BMI) was explored in patients and controls. The relation between the QoL *vs* patients’ characteristics (time since diagnosis, type of hypothyroidism, type of thyroid medication, LT4 dose, serum thyroid parameters, and recent stress/life event) was explored in the patient group.

## Materials and Methods

### Design and ethics

This was an observational study using an online survey (Lime Survey) among Dutch hypothyroid patients and control persons (all anonymous). The protocol was submitted to the Medical Ethical Committee of the University Medical Centre Groningen (UMCG), the Netherlands, and needed no further review (M15.170686, March 6th, 2015). All respondents were informed about the purpose and nature of the study. They voluntarily completed the survey, thereby giving their consent to participate. Data were collected from April 22, 2015, until June 20, 2015.

### Populations

The inclusion criteria for hypothyroid patients were: ≥18 years, diagnosed with hypothyroidism, having thyroid replacement therapy for ≥ 6 months, and for control persons: ≥18 years, no diagnosed thyroid disease. The inclusion criteria were verified by obligatory filtering questions. In The Netherlands, according to the Dutch thyroid guidelines, patients are being diagnosed with overt hypothyroidism only when their TSH concentration is > 4.0 mU/L and their FT4 concentration is < 9 pmol/L [22]. Patients and controls entered the study by self-sampling (TREND guidelines). The survey was disseminated by Dutch patient organizations, posters/flyers in pharmacies and health centers, postings on Twitter/Facebook, a newspaper article, and a TV item. Control subjects were recruited alongside patients (snowballing method), i.e. the accompanying letter asked patients to recruit control persons in their environment (e.g. a partner/friend/family member), to increase control responses with similar sociodemographic characteristics. The text on the leaflets and social media was formulated in an open-ended manner asking hypothyroid patients and control persons for their opinions. The accompanying letter mentioned that the survey was on quality of life in neutral terms.

### Background variables

Respondents voluntarily stated their date of birth, sex, body weight, height, menopause (if female), and comorbidities (self-reporting, SAGER guidelines). To address the impact of comorbidities, we used the M3 comorbidity index. Patients reported their comorbid conditions, and based on ICD-10 guidelines, the M3 index (weighed burden of comorbidities) was calculated [20]. Patients also stated the year of diagnosis of hypothyroidism, their type of hypothyroidism, their thyroid medication, their thyroid hormone replacement dose, and their adherence. If tested, they were invited to provide their most recent TSH, FT4, FT3 values, and TPO antibody positivity/negativity.

### Thyroid parameter reference ranges

This study kept to the Dutch GP reference ranges for serum TSH of 0.4–4.0 mU/L and serum FT4 of 9–24 pmol/L [21]. A free thyronine (FT3) serum reference range of 3.1–6.8 pmol/L was used [23]. TPO antibody (TPO Ab) tests were considered negative if < 60 IE/mL, doubtful when 60–100 IE/mL, and positive if > 100 IE/mL.

### Quality of life questionnaire (ThyPRO)

The quality of life (QoL) was measured by the validated thyroid-specific questionnaire ThyPRO [24]. The Dutch translation of the questionnaire was pretested in a pilot study. The 7 QoL domains of the ThyPRO that were suitable for both patients and controls were: Tiredness (the combination of Fatigue and impaired Vitality), Cognitive problems, Anxiety, Depressivity (depressive feelings), Emotional susceptibility, Social- and Daily life. For the domains Social- and Daily life, the questions were rephrased into “Did you … because of your health” instead of “Has your thyroid disease caused you”, to make answering by both patients and controls possible. Each QoL domain comprised 3–9 questions that were rated on a 0–4 Likert scale. Sum scores per QoL domain were normalized to a 0–100 scale. The ‘Mean QoL’ was the calculated mean of these 7 domains, as a way to represent the overall QoL. Please note that higher ThyPRO scores indicate a greater disruption of the QoL.

### Daily functioning questions (SF36-derived) and statements

Daily functioning was measured using 3 SF36-derived questions on a 1–5 Likert scale, for patients and controls. Patients additionally responded to statements on daily functioning (1–5 Likert scale), and questions about if they experienced the burden of recent stress or life event.

### Hypothyroidism-related symptoms (ThySHI)

Present hypothyroidism-associated symptoms (18 items) were rated on a 0–3 Likert scale by both patients and control persons. Patients additionally rated 8 present items, and retrospectively rated all 26 symptoms at three past time windows: around diagnosis, after 0–1 year, and after 1–3 years (Thyroid Symptom History Inventory, ThySHI).

## Theory and calculations

For mean scores of the ThyPRO, at least 55 % of the questions/domains had to be answered to obtain meaningful results (using mean points in SPSS). Statistical testing for differences between patients and controls was performed using an independent samples *t*-test for numeric variables (ThyPRO; age, body weight, BMI, M3 comorbidity index) and Mann-Whitney tests for ordinal/nominal variables (daily functioning questions, statements, ThySHI, sex, menopausal status). The Mean QoL of patients was divided by the Mean QoL of controls (as %) as a way of expressing patient-control differences. Cohen’s d was additionally calculated to express the extent of patient-control differences in the various QoL domains and the Mean QoL [25]. A multiple linear regression model was used to explore the contribution of possible confounders (background variables) to the Mean QoL in patients and controls. In patients, the relation between the Mean QoL and categorical patient characteristics e.g., serum thyroid categories (low-in range-high), TPO Ab category, types of hypothyroidism, types of thyroid medication, and time since diagnosis was explored by a non-parametric ANOVA (Kruskal-Wallis) with post hoc Bonferroni-tests. Additionally, the relation between the Mean QoL and numeric patient characteristics , e.g. serum thyroid concentrations (TSH, FT4, FT3, and FT3/FT4 ratio), was explored by a linear regression model and Pearson correlation analysis (all in IBM-SPSS version 25, with a p < 0.05 being considered significant).

## Results

### Characteristics of respondents

In total, 1944 persons responded to the survey, of whom 1667 were treated hypothyroid patients and 277 were control persons. All respondents were confirmed to be unique persons who fulfilled all the inclusion criteria. Respondent group sizes, however, may differ per question since all responses were provided voluntarily. The patient- and control groups were not statistically different concerning age, percentage of women in menopause, or weight/BMI (Table 1). The patient group, however, contained more females (92 % *vs* 65 %), had a higher M3 comorbidity index (0.38 *vs* 0.16), and the women had been postmenopausal for a longer time than the control group (Table 1). Respondents (n = 1942) received the survey through patient societies (64 %), social media (7 %), newspaper/TV (5 %), poster/flyer (5 %), doctors (0.4 %), personal network (0.5 %) or other (18 %) (Supplementary Table 1). The patient group (n = 1665) found the survey mainly through patient organizations (75 %) (Supplementary Table 1).

**Table 1:** Characteristics of respondents (hypothyroid patients and control persons).

		Hypothyroid patients		Control persons	
		Number	Percentage	Number	Percentage
Respondents	Total n = 1944	1667		277	
Age	Mean ± SD (ns, p = 0.715)	50.1 ± 12.7 (n = 1464)		49.8 ± 14.3 (n = 241)	
	18–45 years	434	29.6 %	72	29.9 %
	46–65 years	811	55.4 %	124	51.5 %
	66 + years	219	15.0 %	45	18.7 %
Sex (*)	Male	133	8.0 %	95	34.6 %
	Female	1528	92.0 %	180	65.5 %
Menopausal status (for females)	Females in menopause (ns, p = 0.656)	42 % (n = 135)		40 % (n = 1138)	
	Years in menopause (*, p = 0.016)	8.0 ± 7.4 (n = 548)		6.0 ± 5.6 (n = 57)	
Bodyweight (kg)	Mean ± SD (ns, p = 0.808)	78.7 ± 23.0 (n = 1660)		79.5 ± 52.5 (n = 276)	
BMI	Mean ± SD (ns, p = 0.564)	28.9 ± 68.2 (n = 1655)		26.5 ± 19.8 (n = 275)	
Comorbidities top 10	High blood pressure	300	18.0%	27	9.8%
	Menopausal complaints	284	17.1%	14	5.1%
	Vitamin B12 deficiency	242	14.5%	9	3.3%
	Joint/Muscle pain	236	14.2%	8	2.9%
	High cholesterol	228	13.7%	21	7.6%
	Depression	182	10.9%	7	2.5%
	Fibromyalgia	154	9.3%	10	3.6%
	Chronic Fatigue Syndrome	141	8.5%	5	1.8%
	Anxiety disorder	123	7.4%	9	3.3%
	Burnout	117	7.0%	8	2.9%
M3 comorbidity index	Mean ± SD (*, p < 0.001)	0.38 ± 0.42 (n = 1641)		0.16 ± 0.44 (n = 277)	

BMI = body mass index, M3 comorbidity index: see Methods, (ns) = not significant, (*) = significant p < 0.05.

The average time since diagnosis was 10.0 (±9.9) years (Table 2). Most patients reported having Hashimoto’s disease (37.1 %) or did not know the cause of their hypothyroidism (31.3 %). Smaller proportions had other causes of hypothyroidism, such as radioactive-iodine (RAI) ablation of the thyroid for Graves’ disease (8.9 %), resection of the thyroid gland (7.0 %), or congenital- (2.0 %) or central (1.1 %) hypothyroidism (Table 2).

**Table 2:** Further characteristics of treated hypothyroid patients.

		Hypothyroid patients	
		Number	Percentage
Time since diagnosis	Mean ± SD (years)	9.97 ± 9.87 (n = 1612)	
	0.5–5 years	650	40.3 %
	6–15 years	608	37.7 %
	16–25 years	251	15.6 %
	>25 years	103	6.4 %
Type of hypothyroidism	Hashimoto’s disease	619	37.1 %
	RAI for Graves’ disease	148	8.9 %
	Resection thyroid gland malignant	56	3.4 %
	Resection thyroid gland non-malignant	60	3.6 %
	Congenital hypothyroidism	34	2.0 %
	Pituitary tumor/dysfunction	19	1.1 %
	I don’t know	521	31.3 %
	Other cause	210	12.6 %
Type of thyroid medication	LT4 only	1221	80.5 %
	LT4 + LT3	118	7.8 %
	LT4 + DTE	79	5.2 %
	DTE	91	6.0 %
	LT4 + LT3 + DTE	3	0.2 %
	LT3 only	2	0.1 %
	LT3 + DTE	2	0.1 %
Dose LT4	Mean ± SD (mcg)	123.0 ± 45.5 (n = 1159)	
	Median (mcg), IQR (25–75 %)	125.0 (100.0–150.0)	
	Mean ± SD (mcg/kg)	1.60 ± 0.58 (n = 1157)	
Adherence to therapy	Taking the prescribed dose: Always	1406	95 %
	On empty stomach: Always	1390	90 %
TSH (serum)	Median (IQR 25–75 %) (mU/L)	0.90 (0.23–2.20)	
	Mean ± SD (mU/L)	1.58 ± 2.05 (n = 926)	
	Low (<0.4 mU/L)	308	33.2 %
	In range (0.4–4 mU/L)	533	57.5 %
	High (>4 mU/L)	86	9.3 %
FT4 (serum)	Median, IQR (25–75 %) (pmol/L)	17.00 (14.30–20.00)	
	Mean ± SD (pmol/L)	17.18 ± 4.48 (n = 905)	
	Low (<9 pmol/L)	21	2.3 %
	In range (9–24 pmol/L)	828	91.4 %
	High (>24 pmol/L)	57	6.3 %
FT3 (serum)	Median, IQR (25–75 %) (pmol/L)	2.67 (1.60–4.40)	
	Mean ± SD (pmol/L)	3.39 ± 3.38 (n = 320)	
	Low (<3.1 pmol/L)	168	52.3 %
	In range (3.1–6.8 pmol/L)	144	44.9 %
	High (>6.8 pmol/L)	9	2.8 %
FT3/FT4 ratio	Mean ± SD	0.23 ± 0.24 (n = 313)	
	Median, IQR (25–75 %)	0.18 (0.10–0.29)	
TPO antibodies	Negative (<60 IE/mL)	37	23.4 %
	Doubtful (60–100 IE/mL)	6	3.8 %
	Positive (>100 IE/mL)	115	72.8 %

RAI = radioactive iodine treatment, LT4 = levothyroxine, LT3 = liothyronine, DTE = desiccated thyroid extract.

TSH = thyroid stimulating hormone, FT4 = free thyroxine, FT3 = free liothyronine, TPO = thyroid peroxidase.

The vast majority of patients used LT4-only therapy (80 %, n = 1221), whereas 8 % used LT4 + LT3 (n = 118), 6 % used desiccated thyroid extract (DTE) (n = 91), and 5 % used LT4 + DTE (n = 79). Patients reported high adherence to their thyroid replacement therapy, in terms of taking the prescribed dose and fasting state (Table 2). The median LT4 dose taken was 125 mcg (mean 123 mcg) and the mean LT4 dose/kg was 1.6 mcg (Table 2).

The median TSH concentration in treated hypothyroid patients was 0.90 mU/L (mean 1.58 mU/L) (Table 2). In 58 % of patients the TSH level was within range (0.4–4 mU/L). One-third (33 %) had serum TSH concentrations below 0.4 mU/L. The median FT4 concentration was 17.0 pmol/L (mean 17.2 pmol/L), and almost all patients (91 %) had FT4 levels within the reference range (9–24 pmol/L) (Table 2). The median FT3 concentration was 2.67 pmol/L (mean 3.39 pmol/L), with 45 % of patients within the range (3.1–6.8 pmol/L). About half of the patients (52 %) had a low FT3 concentration (<3.1 pmol/L) (Table 2). The mean FT3/FT4 ratio was 0.23. TPO antibody (TPO Ab) positivity (>100 IE/mL) was reported by 73 % (Table 2). As compared to LT4 users, patients using LT4 + LT3 had lower serum TSH and FT4 concentrations with similar FT3 levels. Patients using LT4 + DTE had similar TSH, FT4, and FT3 concentrations as LT4 users. Patients using DTE had similar serum TSH, lower FT4 and higher FT3 concentrations *vs* LT4 users (Supplementary Fig. 2).

### Quality of life (ThyPRO)

**Fig. 1:**
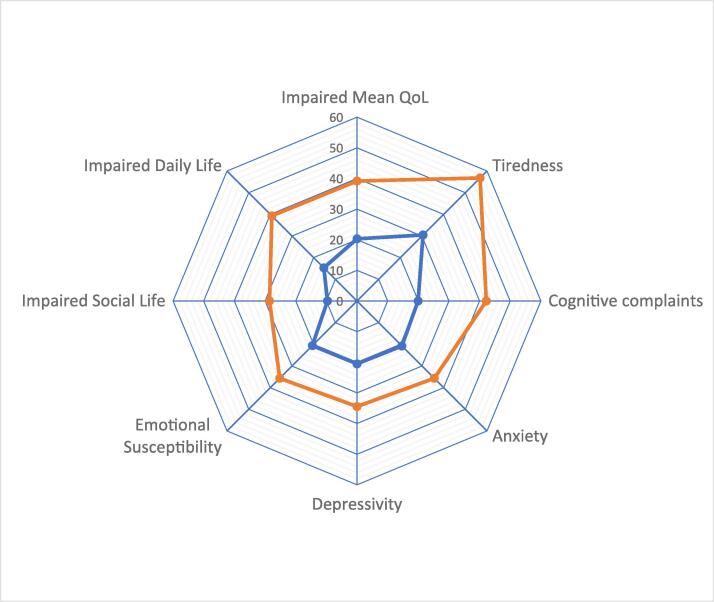
Quality of Life (QoL, using the ThyPRO, expressed as means) in treated hypothyroid patients (orange, n = 1195) and controls (blue, n = 240). Mean QoL and all domains were significantly more impaired in hypothyroid patients as compared to controls (p < 0.001). Orange = hypothyroid patients (treated with thyroid hormone), Blue = controls. (For interpretation of the references to color in this figure legend, the reader is referred to the web version of this article.)

The Mean QoL in treated hypothyroid patients (n = 1195) was significantly and large-sized impaired as compared to controls (n = 236) (ThyPRO score 39.2 and 20.3, +93 %, for patients and controls resp., Cohen’s d = 1.04, p < 0.001) (Fig. 1). The QoL domains Tiredness, Cognitive complaints, Emotional Susceptibility, Social- and Daily life showed large-sized impairments (Cohen’s d > 0.8), and medium-sized impairments (Cohen’s d 0.5–0.8) were observed for Anxiety and Depressivity (Fig. 1, Table 3).

**Fig. 2:**
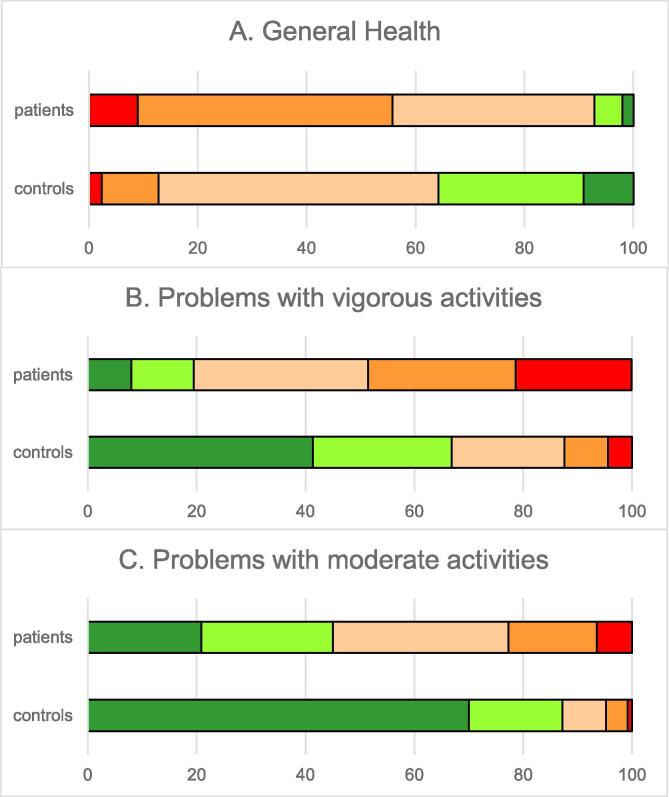
Daily Functioning of treated hypothyroid patients (n = 1206) and controls (n = 250) (number of respondents, as % of total response). Treated hypothyroid patients had significantly reduced daily functioning as compared to controls on all 3 items (p < 0.001). Red = worst functioning, green = best functioning. (For interpretation of the references to color in this figure legend, the reader is referred to the web version of this article.)

**Table 3:** ThyPRO scores (0–100) for control persons and hypothyroid patients on 7 domains and the Mean QoL, with patient/control percentages, p-values and Cohen’s d.

	Hypothyroid Patients			Control persons			Statistics			
	n	Mean (0-100)	SD	n	Mean (0–100)	SD	Mean QoL (patient)/Mean QoL (control), as %	p-value, two-tailed *t*-test	Cohen’s d for independent samples	Interpretation Cohen’s d effect size*
Impaired Mean QoL	1195	39.2	18.5	236	20.3	15.6	193 %	p < 0.005	1.04	Large effect
1. Tiredness	1186	56.8	22.5	233	30.5	19.9	186 %	p < 0.005	1.19	Large effect
2. Cognitive complaints	1168	42.2	24.3	226	19.9	18.2	212 %	p < 0.005	0.95	Large effect
3. Anxiety	1160	35.7	22.7	224	20.7	19.0	172 %	p < 0.005	0.67	Medium effect
4. Depressivity	1155	34.4	22.6	225	20.5	18.0	168 %	p < 0.005	0.63	Medium effect
5. Emotional Susceptibility	1150	35.7	18.8	224	20.6	16.4	173 %	p < 0.005	0.82	Large effect
6. Impaired Social Life	1138	28.5	21.8	166	9.6	15.9	297 %	p < 0.005	0.90	Large effect
7. Impaired Daily Life	1119	39.3	26.4	157	15.3	22.7	257 %	p < 0.005	0.92	Large effect

*Cohen’s d 0.5–0.8: medium-sized effect, d ≥ 0.8: large effect.

The Mean QoL of respondents (patients and controls) was not associated with age, sex, body weight, BMI, stress/life event, or menopausal status. The M3 comorbidity index significantly decreased the patient-control QoL difference by 14 %, leaving the QoL difference between patients and controls still large and significant after adjustment (Beta with M3 index = 18.9 *vs* Beta without M3 index = 16.3).

In the patient group, the Mean QoL was not significantly related to TSH-, FT4-, FT3- categories (low-in range-high), TPO Ab categories (negative-doubtful-positive), types of hypothyroidism, nor to most medication types (LT4 vs LT4 + LT3 and LT4 + DTE). Only DTE users reported significantly (22 %) less impairment of Mean QoL than LT4 users (Mean QoL 31.0 *vs* 39.8 resp.). Patients having been hypothyroid for more than 5 years had significantly less impairment of Mean QoL than patients with a disease duration < 5 years (Table 4). The linear regression model revealed no relation between serum FT4- or FT3-concentrations and Mean QoL, but a significant (p = 0.010) though very weak positive relation between TSH levels and Mean QoL (Beta 0.79 with Beta Intercept 37.94, Pearson correlation coefficient R^2^ = 0.007) (Supplementary Fig. 3). We found no relation between the Mean QoL and the LT4 dose used.

**Table 4:** Quality of Life (Mean Qol, ThyPRO 1–100 scores) of hypothyroid patients in serum TSH-, FT4-, FT3- and TPO Abs categories, types of hypothyroidism, types of thyroid medication, and duration of hypothyroidism.

	Category	n	Mean QoL	SD
TSH category	Low (<0.4 mU/l)	293	38.5	17.6
	Normal (0.4–4 mU/l)	510	38.3	18.1
	High (>4 mU/l)	78	42.7	18.4
	ANOVA TSH categories p = 0.135 (ns)			
FT4 category	Low (<9 pmol/l)	19	43.3	22.6
	Normal (9–24 pmol/l)	790	38.8	17.9
	High (>24 pmol/l)	55	41.5	18.4
	ANOVA FT4 categories p = 0.362 (ns)			
FT3 category	Low (<3.1 pmol/l)	158	39.2	17.5
	Normal (3.1–6.8 pmol/l)	142	38.0	19.7
	High (>6.8 pmol/l)	7	41.7	24.7
	ANOVA FT3 categories p = 0.776 (ns)			
TPO Ab category	Negative (<60 IE/ml)	34	42.9	16.4
	Doubtful (60–100 IE/ml)	6	49.4	17.4
	Positive (>100 IE/ml)	114	43.2	18.3
	ANOVA TPO Abs categories p = 0.702 (ns)			
Type of hypothyroidism	Hashimoto’s disease	458	39.9	19.0
	RAI for Graves	111	40.6	18.5
	Resection malignant	41	41.3	17.6
	Radiation	2	46.3	5.4
	Resection non-malignant	64	40.1	14.97
	Congenital hypothyroidism	28	41.8	18.8
	Pituitary disorder	11	42.5	17.0
	Don’t know	369	38.1	18.9
	ANOVA Type of hypothyroidism p = 0.771 (ns)			
Type of thyroid medication	LT4	992	39.8	18.1
	LT4 + LT3	86	40.0	15.8
	LT4 + DTE	63	40.3	21.0
	DTE (*)	77	31.0	21.2
	ANOVA Type of thyroid medication p < 0.0005 (*)			
Duration of hypothyroidism	0.5–5 yrs	468	41.5	17.9
	6–15 yrs (*)	436	37.3	19.1
	16–25 yrs (*)	184	38.3	19.4
	>26 yrs	80	38.7	18.6
	ANOVA Duration of illness p = 0.006 (*)			

TSH = thyroid stimulating hormone, FT4 = free thyroxine, FT3 = free liothyronine, TPO Ab = thyroid peroxidase antibodies, RAI = radioactive iodine treatment, LT4 = levothyroxine, LT3 = liothyronine, DTE = desiccated thyroid extract, (ns) = not significant-, (*) = significant effect (p < 0.05). DTE differed from LT4, LT4 + LT3, LT4 + DTE (p < 0.005, p = 0.003, p = 0.011 resp.). Duration 6–15 yrs and 16–25 yrs differed from 0.5 to 5 yrs (p = 0.004, p = 0.43 resp).

### Daily functioning (SF36-derived items and statements)

General health was significantly impaired (-38 %) in hypothyroid patients as compared to controls (p < 0.001). Hypothyroid patients had + 64 % and + 77 % more trouble with vigorous- and moderate physical efforts than control persons (p < 0.001) (n = 1206) (Fig. 2).

About three-quarters of treated hypothyroid patients stated their family life (75 %), private/social life (79 %), and professional life (72 %) was ‘somewhat’ to ‘very much’ impacted by their hypothyroidism (n = 1201) (Table 5). A large majority of patients indicated that they suffered from complaints despite thyroid replacement therapy (78 %) and despite ‘in-range’ thyroid blood values (79 %, n = 1194). Three-quarters of patients said they did not feel their own self again (75 %) and would like to have better treatment options for their hypothyroidism (75 %) (n = 1194) (Table 5).

**Table 5:** Statements Daily Life (% agreement and % disagreement from treated hypothyroid patients).

	n	Percentage of patients that (somewhat-very much) agree: % YES	Percentage of patients that (somewhat-very much) disagree: % NO
My family life has been impacted by my hypothyroidism.	1201	75 %	25 %
My private/social life has been impacted by my hypothyroidism.	1201	79 %	21 %
My professional life has been impacted by my hypothyroidism.	1200	72 %	28 %
I suffer from complaints despite my thyroid medication.	1194	79 %	10 %
When the doctor says my blood values are good I’m feeling fine.	1194	7 %	78 %
I feel like my own self again.	1192	10 %	75 %
I would like to have better treatment options for hypothyroidism	1194	75 %	7 %

N.B. The neutral answers (no agreement/no disagreement) were left out.

### Hypothyroidism-associated symptoms (ThySHI)

#### Eighteen symptoms (in the present) of patients and controls

**Fig. 3:**
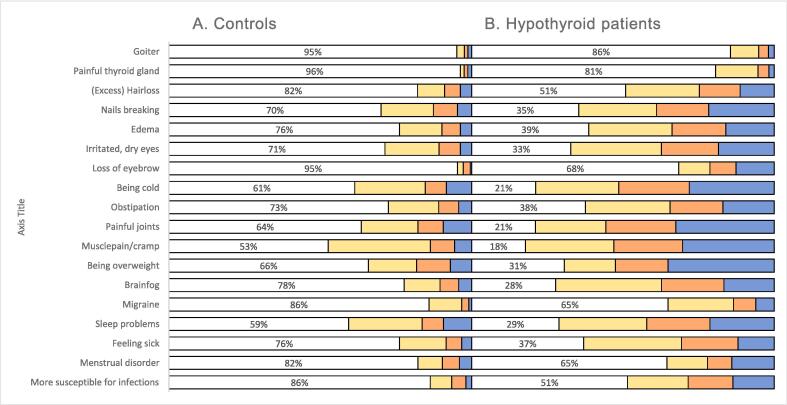
Symptoms (at present, 0–3 scale) in treated hypothyroid patients (right panel, n = 940) and controls (left panel, n = 214), as a percentage of the population. White = score 0 (absent), yellow = score 1(little), orange = score 2 (moderate) and blue = score 3 (intense). All symptoms were significantly more intensely present in treated hypothyroid patients than in controls (p < 0.001). (For interpretation of the references to color in this figure legend, the reader is referred to the web version of this article.)

Treated hypothyroid patients rated on average 2.3 times more frequent and 2.8 times more intense thyroid-related symptoms than control persons (see Fig. 3 for frequencies and Table 6 for means). All separate 18 symptoms assessed (ThySHI) were significantly more present in patients than in controls (all items p < 0.001). On average 24 % of the control group and 54 % of the patient group reported hypothyroidism-associated symptoms (i.e. had a score ≥ 1) (Fig. 3). Symptoms that were most intensely present in patients as compared to controls (i.e. mean of patients / mean of controls) were: loss of eyebrow (8 times more intense), getting sick/infections faster/more often and painful thyroid gland (about 4 times more intense), brain fog, migraine, edema, feeling sick, irritated/dry eyes, (excess) hair loss, and being overweight (about 3 times more intense) (Table 6).

**Table 6:** Hypothyroidism-associated symptoms (ThySHI) in (treated) hypothyroid patients 1. Before/around diagnosis, 2. After 0–1 year, 3. After 1–3 years and in the present (0–3 scores), in blue are the hypothyroid patients, and in green are the controls. Yellow bars indicate how much worse patient’s symptoms were as compared to controls (mean patients/mean controls as a percentage). Purple bars show the improvement (+) or worsening (-) in the present as compared to the time around diagnosis.

#### Twenty-six symptoms of patients (ThySHI present and past)

The 10 most intense symptoms of hypothyroid patients (in the present) were: fatigue > painful joints > muscle pain/cramp > being cold > being overweight > impaired functioning > decreased libido > concentrations problems > sleep problems > brain fog (Table 6).

All 26 symptoms that were rated (ThySHI) to be present around diagnosis (100 %), were still present after 0–1 year (92 %), 1–3 years (83 %), and continued into the present (84 %) (Table 6).

## Discussion

### QoL, daily functioning and symptoms in patients and controls

This study shows major reductions in QoL and daily functioning, and almost 3-times more intense hypothyroidism-associated symptoms in treated hypothyroid patients *vs* control persons, despite thyroid replacement therapy and serum TSH/FT4 concentrations predominantly within the reference range. These results are substantiated by a large number of patients and an extensive survey. The QoL impairments are found across all 7 domains considered but are most prominent for Cognition and Tiredness (which includes fatigue and vitality questions), which is in line with other studies showing fatigue, reduced well-being and cognition in treated hypothyroid patients [8], [9], [26].

Comorbidities, as expressed by the M3 index, are more abundant in patients than controls, but explained only 14 % of the QoL reduction in patients. This means that the majority of QoL reduction is not accounted for by comorbidities and is most likely related to (consequences of) the thyroid disease itself. This is supported by the finding that 84 % of the symptom load persisted since the time around diagnosis. A limitation here could be recall bias as this was rated retrospectively. Of note are the relatively high incidences of burnout in the patient population. According to Dutch guidelines, the diagnosis burnout cannot be made when a patient has a thyroid disease. Comorbidities were self-reported in this survey. Therefore, the high prevalence of burnout is probably reflecting misdiagnoses.

Among the patients in this study, three-quarters suffer from complaints despite thyroid medication and in-range thyroid blood values and wish for better treatment, which is much higher than the 5–15 % reported in the literature [1], [6]. The data underlying the 5–15 %, however, are unclear and the exact proportion of treated hypothyroid patients with persisting complaints may actually not be well-known. The high percentage of patients with persisting complaints in this study could have been attributed to the open exploratory research design depending on voluntary participation in the survey and the recruitment through patient organizations, which may have led to overrepresentation of patients with complaints (selection bias). Comparing these ThyPRO scores of treated hypothyroid patients with literature revealed that our data was similar or only modestly higher (meaning worse QoL) than other studies (Supplementary Fig. 4) [27], [24], [28], [29]. The recruitment of controls through patients (snowballing) may also have led to selection bias, but our ThyPRO scores were the same as in the study of Cramon [27]. Therefore, it seems there is no important selection bias in the control group, and perhaps some selection bias towards a lower QoL in the patient group, possibly also because all comorbidities were allowed in this study. Caution is still needed when extrapolating the exact percentages of this study to the entire hypothyroid population.

The most intense symptoms reported by patients are fatigue, followed by painful muscles and joints, and many other classic hypothyroid symptoms, in line with other studies [30], [31]. Notably, the patients in this study did not report significantly higher body weight or BMI but did rate themselves more often as “being overweight”, than controls. There were, however, large variations in the body weight and BMI values. Weight gain or obesity have a complicated relationship with thyroid hormones, and LT4 treatment only modestly reduces body weight in hypothyroid patients [17]. The patient group reported having more edema and obstipation than controls, which could possibly contribute to the contradictory results on body weight/BMI (objective parameters) *vs* reporting being overweight (subjective parameter).

Patient-reported outcomes (PROs) become more important in the monitoring of many diseases, including hypothyroidism, to obtain a better understanding of the impact of illness on the patient, thereby facilitating communication with the physician and leading to co-decision of a better treatment plan.

This study adds to the accumulating data that a considerable number of hypothyroid patients on LT4 (or other thyroid) replacement therapy experience (severe) persisting complaints despite TSH/FT4 concentrations being (largely) in range. Together with the growing number of hypothyroid patients, we see a significant clinical problem for which no adequate therapy exists today [32].

### QoL vs various thyroid replacement therapies

In our study, the various thyroid replacement therapies i.e., LT4, LT4 + LT3, LT4 + DTE, revealed similar QoL. Several *meta*-analysis studies showed that LT4 + LT3 combination therapy generally not improved the QoL but did enhance the patient’s preference [33], [34], [35]. Results of new LT4 + LT3 combination trials and the development of new sustained-release or tissue-directed T3 formulations are awaited [34].

Desiccated thyroid hormone (DTE) users reported a superior QoL compared to LT4 treatment. This differs from studies where DTE users had a similar QoL as LT4 users, although DTE was preferred over LT4 [36], [37]. Notably, DTE users in our study reported higher FT3 concentrations (with similar TSH values) than LT4 users, in line with the Shakir study, which may be key to their superior QoL [37].

### QoL vs serum thyroid parameters

The self-reported, most recent, serum TSH/FT4 serum concentrations of patients were predominantly in range (58 % of TSH- and 91 % of FT4 concentrations). The lower TSH values (<0.4 mU/L) seen in one-third (32 %) of patients, are often needed to obtain sufficient FT4 concentrations under LT4 treatment, and do not necessarily need to be regarded as inadequately dosed [38], [39], [40], [41]. In the Parle study, 21 % of treated hypothyroid patients had a TSH value below the reference range, which is somewhat less than in our study [42]. In our study, 52 % of patients had low FT3 concentrations, which is much more than the 15 % reported by Gullo in athyreotic LT4-treated patients [43]. LT4 monotherapy has been associated with low serum FT3 levels [41]. The FT3/FT4 ratio in our study (=0.23) was similar to LT4-treated hypothyroid patients in the study of Alevizaki (=0.22) and were both much lower than that of healthy people (=0.32) [44]. Low serum FT3 levels and low FT3/FT4 ratios with LT4 treatment would suggest tissue hypothyroidism, which could - at least in part - explain persisting hypothyroid symptoms and would contradict the hypothesis that local deiodinases always convert T4 into sufficient T3 levels [30], [45]. Several authors believe that, although LT4 replacement restores (hypothalamic) TSH concentrations, it may in fact not fully restore euthyroidism at the tissue or receptor level [30], [41], [46], [47], [48], [49]. Our data might fit with this ‘low T3 ' hypothesis to explain persisting symptoms, since low serum FT3 concentrations were reported concurrent with low FT3/FT4 ratios. Given the relatively low number of patients who had their FT3 level tested (as this is not part of the guidelines in The Netherlands) our study was underpowered to address this issue in a reliable way.

In this study, the QoL was not found to be related to any of the serum thyroid parameters (TSH, FT4, FT3, FT3/FT4 ratio), nor the LT4 dose taken. There was, however, a very weak though significant correlation between TSH concentrations and QoL. The clinical impact of this would be small, given that the QoL in the TSH categories (low-normal-high) was similar. Other studies have reported varying relationships between serum thyroid parameters and QoL-like variables under LT4 treatment: well-being was found to be related to FT4 (not FT3), whereas residual hypothyroid symptoms were found to be related to FT3, (and less to FT4 and TSH) [50], [51]. Others found no association between QoL and any serum thyroid parameter or LT4 dose [52], [53]. The relation between QoL and serum TSH/FT4/FT3 concentrations or LT4 dose, thus, remains uncertain. Our results are in line with other studies, suggesting the TSH concentration may be of limited use in predicting clinical- and (peripheral) thyroid status under LT4 replacement therapy [12], [43], [49], [54], [55]. A limitation here is that TSH-, FT4, and FT3 concentrations of patients were self-reported and not measured simultaneously with the survey.

### Heterogeneity in the pathophysiology of hypothyroidism

Hypothyroidism is a prevalent disorder that is heterogenous both in etiology and severity, comprising e.g. autoimmune- and/or iatrogenic factors, polymorphisms of deiodinase- or transporter genes, individual differences in the regulation (set-point) of the hypothalamus-pituitary-thyroid (HPT) axis, individual differences in the HPT-response to LT4 treatment, (the extent of) thyroid resistance, residual thyroid gland function, and different durations of ongoing hypothyroidism [26], [32], [56], [57], [39], [58], [59], [60]. Unique combinations of these factors, and likely many that are as yet unknown, compose the pathophysiology of an individual hypothyroid patient. As such, hypothyroidism can be considered a complex heterogenic condition and it is therefore not surprising that LT4 monotherapy does not relieve thyroid symptoms in all patients.

## Conclusions

This extensive online survey with a large number of hypothyroid patients in the Netherlands showed major impairments of quality of life and daily functioning, and an almost 3-fold higher symptom load *vs* controls, despite LT4 replacement therapy and predominantly in-range TSH/FT4 concentrations. Our study indicated that clinical serum thyroid testing of thyroid status was not related to patient’s experiences about their QoL. We see a medical need in a large group of hypothyroid patients where current standard LT4 replacement therapy does not suffice. Caregivers and patients should be aware that hypothyroid symptoms may linger and disrupt QoL and daily functioning despite following current guidelines.

We would recommend future research into the origin of persisting complaints and developing innovative treatment modalities for hypothyroidism. Suggestions for improved therapy would include optimizing thyroid (i.e. improved LT4 + LT3 combinations) replacement therapy to individual standards, utilizing sustained release T3 formulations, rethinking the controlled use of DTE, and developing innovative thyroid treatments beyond replacement therapy e.g. reducing thyroid autoimmunity, pharmacologically targeting deiodinases or thyroid hormone receptors, or transplanting thyroid organoids/stem cells in order to reinstate thyroid signaling in as many as possible hypothyroid patients [30], [65], [66], [61], [62], [63], [64]. Thyroid patient monitoring could be enhanced by establishing valid functional biomarkers of (peripheral) thyroid status, rethinking individual reference values for thyroid parameters, rethinking the definition of euthyroidism, and applying patient-reported outcomes (PROs) on a regular basis [62], [63], [67], [27].

## CRediT authorship contribution statement

**Ellen Molewijk:** Conceptualization, Data curation, Formal analysis, Investigation, Methodology, Project administration, Software, Supervision, Validation, Visualization, Writing – original draft, Writing – review & editing. **Eric Fliers:** Conceptualization, Methodology, Supervision, Writing – original draft, Writing – review & editing, Validation. **Koen Dreijerink:** Conceptualization, Methodology, Supervision, Validation, Writing – original draft, Writing – review & editing. **Ad van Dooren:** Conceptualization, Methodology, Supervision, Writing – original draft, Writing – review & editing. **Rob Heerdink:** Data curation, Formal analysis, Investigation, Methodology, Supervision, Validation, Writing – original draft, Writing – review & editing.

## Declaration of competing interest

The authors declare that they have no known competing financial interests or personal relationships that could have appeared to influence the work reported in this paper.

## Appendix A Supplementary data

Supplementary data 1 Supplementary Figure 1. Survey found through various sources (patients and controls, n=1942). Sky blue=social media, navy blue=patient organizations, red=newspaper/TV, orange=poster/flyer, green=other.

Supplementary data 2 Supplementary Figure 2. Thyroid parameters (A. Serum TSH, B. Serum FT4, C. Serum FT3) with different thyroid medications: LT4 (n=158), LT4+LT3 (n=46), LT4+DTE (n=37), DTE (n=39). LT4=levothyroxine, LT3=liothyronine, DTE=desiccated thyroid extract. Significant differences (p<0.05) in panel A. Serum TSH: LT4 vs LT4+LT3, in panel B. Serum FT4: LT4 vs LT4+LT3 and DTE; LT4+DTE vs DTE, and in panel C. Serum FT3: LT4 vs DTE.

Supplementary data 3 Supplementary Figure 3. TSH concentrations (mU/L) versus Mean QoL (QoL impairment) scatterplot, y=37.5+0.79x, p=0.010, R square=0.007, p= 0.010.

Supplementary data 4 Supplementary Figure 4. Comparing ThyPRO values with the literature. The Mean QoL for 7 domains (Tiredness, Cognitive complaints, Anxiety, Depressivity, Emotional Susceptibility, Impaired Social Life, Impaired Daily Life) was used for patient data. The Mean QoL of 5 domains Tiredness, Cognitive complaints, Anxiety, Depressivity, Emotional Susceptibility) was used for control data.
