# Supplementary figures and images for: Quality of life, daily functioning, and symptoms in hypothyroid patients on thyroid replacement therapy: A Dutch survey
Source: J Clin Transl Endocrinol. 2024 Feb 2;35:100330. doi: 10.1016/j.jcte.2024.100330 (PMC10864335; doi:10.1016/j.jcte.2024.100330)

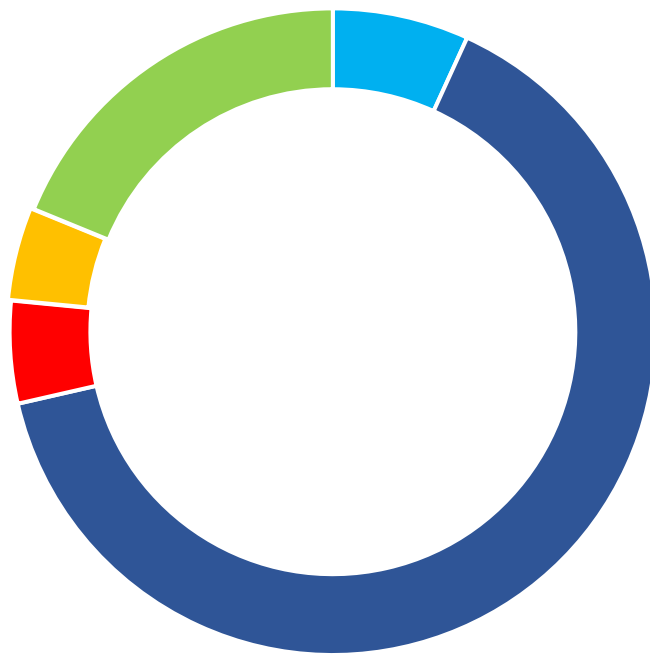

- social media
- patient organization
- newspaper, tv
- poster/flyer HU
- other

Supplement: Supplementary data 1 — Supplementary Figure 1. Survey found through various sources (patients and controls, n=1942). Sky blue=social media, navy blue=patient organizations, red=newspaper/TV, orange=poster/flyer, green=other. [file mmc1.pdf]

### A. Serum TSH

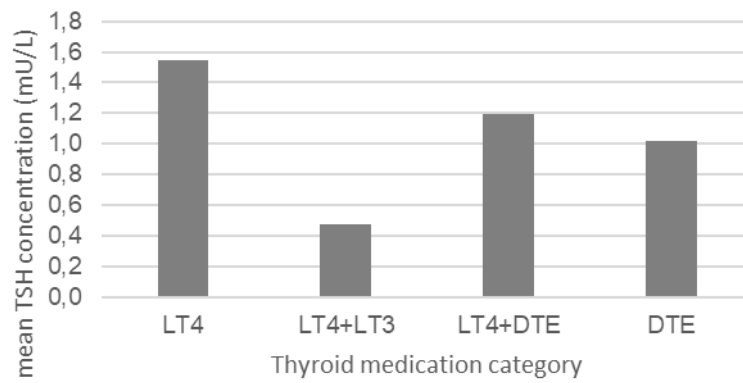

### B. Serum FT4

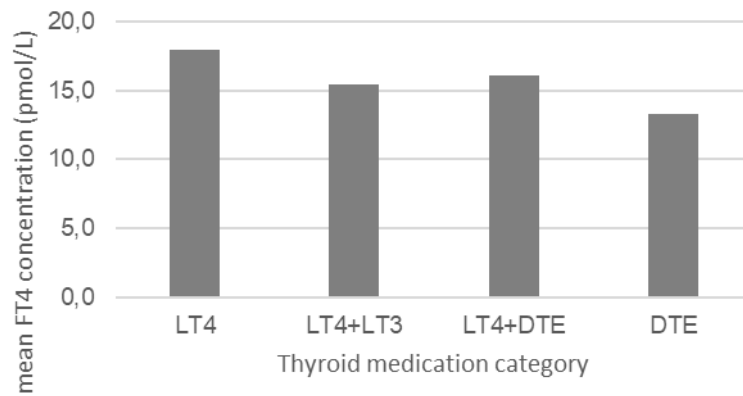

### C. Serum FT3

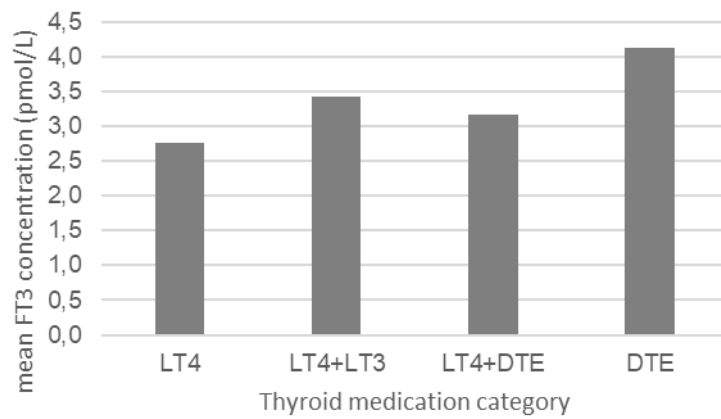

Supplement: Supplementary data 2 — Supplementary Figure 2. Thyroid parameters (A. Serum TSH, B. Serum FT4, C. Serum FT3) with different thyroid medications: LT4 (n=158), LT4+LT3 (n=46), LT4+DTE (n=37), DTE (n=39). LT4=levothyroxine, LT3=liothyronine, DTE=desiccated thyroid extract. Significant differences (p<0.05) in panel A. Serum TSH: LT4 vs LT4+LT3, in panel B. Serum FT4: LT4 vs LT4+LT3 and DTE; LT4+DTE vs DTE, and in panel C. Serum FT3: LT4 vs DTE. [file mmc2.pdf]

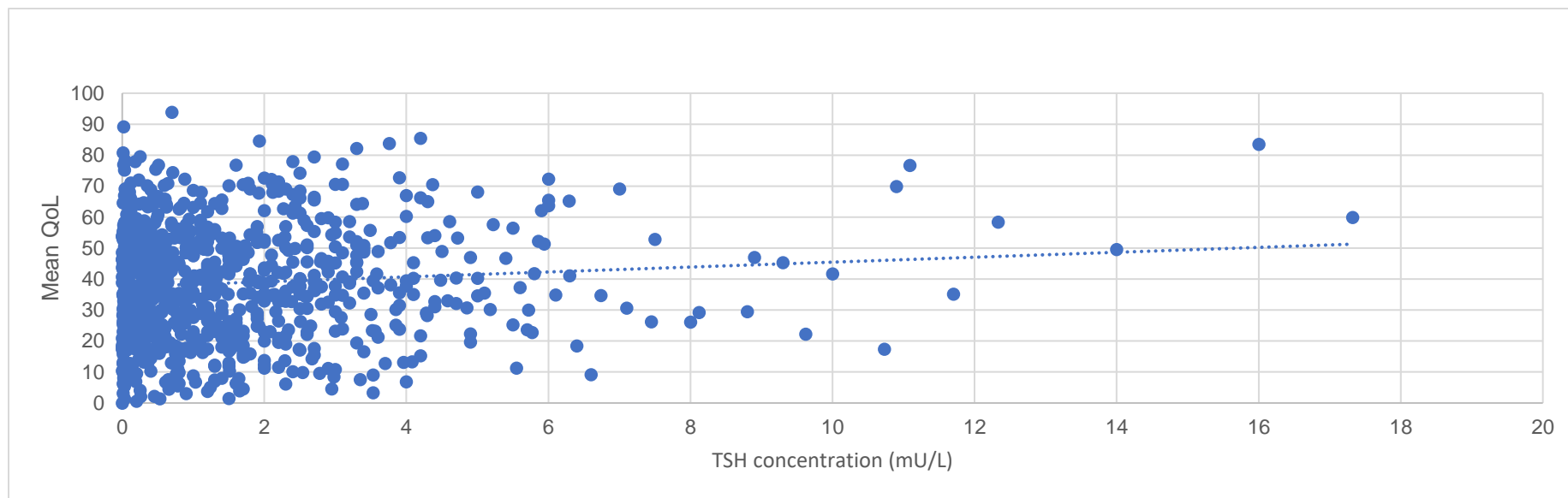

Supplement: Supplementary data 3 — Supplementary Figure 3. TSH concentrations (mU/L) versus Mean QoL (QoL impairment) scatterplot, y=37.5+0.79x, p=0.010, R square=0.007, p= 0.010. [file mmc3.pdf]

A. Hypothyroid Patients

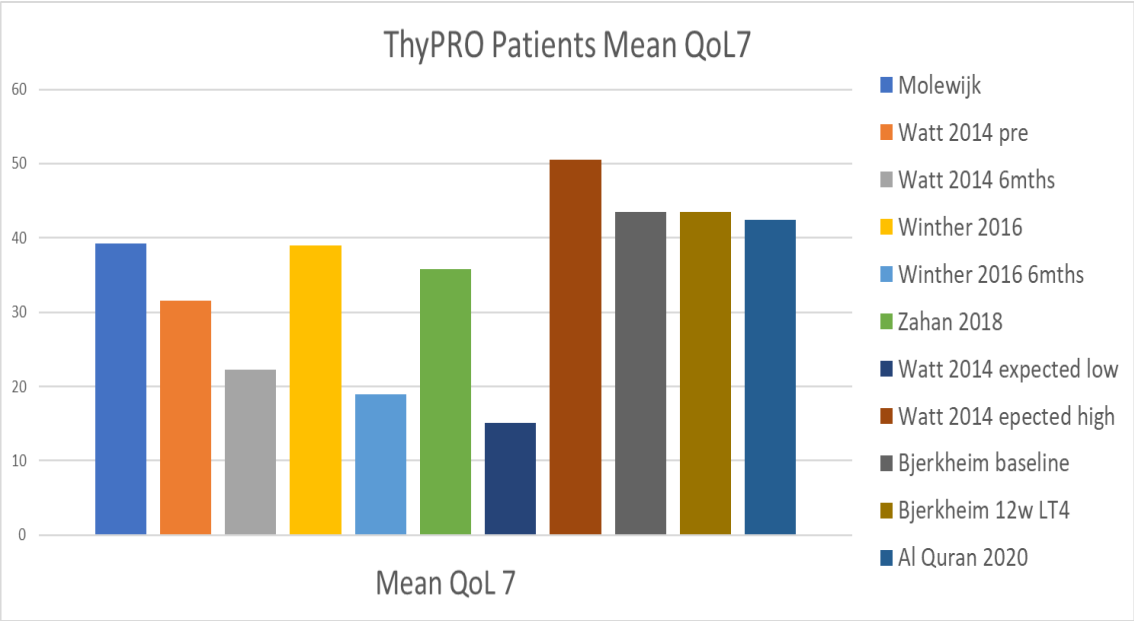

B. Control groups

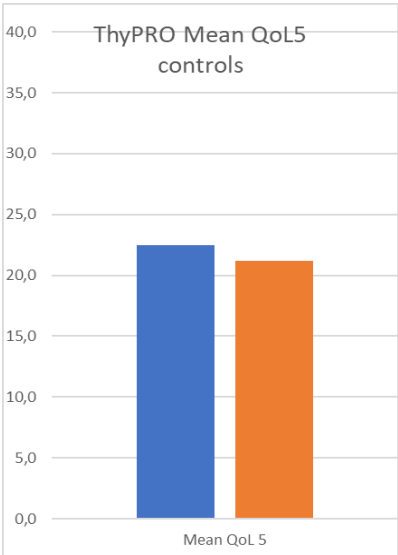

Supplement: Supplementary data 4 — Supplementary Figure 4. Comparing ThyPRO values with the literature. The Mean QoL for 7 domains (Tiredness, Cognitive complaints, Anxiety, Depressivity, Emotional Susceptibility, Impaired Social Life, Impaired Daily Life) was used for patient data. The Mean QoL of 5 domains Tiredness, Cognitive complaints, Anxiety, Depressivity, Emotional Susceptibility) was used for control data. [file mmc4.pdf]
