# Supplementary material for: Quality of life, daily functioning, and symptoms in hypothyroid patients on thyroid replacement therapy: A Dutch survey
Source: J Clin Transl Endocrinol. 2024 Feb 2;35:100330. doi: 10.1016/j.jcte.2024.100330 (PMC10864335; doi:10.1016/j.jcte.2024.100330)
Supplement: Supplementary data 5 [file mmc5.docx]

Supplementary Table 1. Source of receiving the survey.

|  | Control persons | | Hypothyroid patients | | All respondents | |
| --- | --- | --- | --- | --- | --- | --- |
|  | n | % | n | % | n | % |
| social media | 9 | 3% | 123 | 7% | 132 | 7% |
| patient organization | 4 | 1% | 1246 | 75% | 1250 | 64% |
| newspaper, tv | 0 | 0% | 99 | 6% | 99 | 5% |
| poster/flyer | 0 | 0% | 76 | 5% | 90 | 5% |
| personal network | 14 | 5% | 10 | 1% | 10 | 0.5% |
| doctor | 0 | 0% | 7 | 0.4% | 7 | 0.4% |
| other | 250 | 90% | 104 | 6% | 354 | 18% |
| Total | 277 | 100% | 1665 | 100% | 1942 | 100% |
